# Characterization, Antioxidant Capacity, and In Vitro Bioaccessibility of Ginger (*Zingiber officinale Roscoe*) in Different Pharmaceutical Formulations

**DOI:** 10.3390/antiox14070873

**Published:** 2025-07-17

**Authors:** Lucía Plana, Javier Marhuenda, Raúl Arcusa, Ana María García-Muñoz, Pura Ballester, Begoña Cerdá, Desirée Victoria-Montesinos, Pilar Zafrilla

**Affiliations:** Faculty of Pharmacy and Nutrition, Universidad Católica de Murcia (UCAM), Campus de los Jerónimos, Guadalupe, 30107 Murcia, Spain; lcplana@ucam.edu (L.P.); jmarhuenda@ucam.edu (J.M.); rarcusa@ucam.edu (R.A.); pballester@ucam.edu (P.B.); bcerda@ucam.edu (B.C.); dvictoria@ucam.edu (D.V.-M.); mpzafrilla@ucam.edu (P.Z.)

**Keywords:** *Zingiber officinale*, phenolic content, nutraceuticals, bioaccessibility, pharmaceutical formulations, in vitro digestion

## Abstract

Ginger (*Zingiber officinale Roscoe*) has been widely recognized for its antioxidant properties, primarily attributed to its phenolic compounds such as gingerols and shogaols. However, limited data exist regarding how different pharmaceutical forms influence the bioaccessibility and antioxidant efficacy of these compounds. This study aimed to evaluate the antioxidant capacity and bioaccessibility of ginger in different pharmaceutical forms—capsules (20 mg, 40 mg, and 80 mg), a pure powdered extract, and a liquid formulation—standardized to ≥6% gingerols. The phenolic profile of each formulation was characterized using HPLC-DAD (High-Performance Liquid Chromatography with Diode Array Detection), followed by the evaluation of antioxidant capacity through DPPH (2,2-Diphenyl-1-picrylhydrazyl) and ORAC (Oxygen Radical Absorbance Capacity) assays, and the assessment of bioaccessibility via an in vitro digestion model. The results demonstrated that antioxidant activity was positively correlated with extract concentration and was highest in the liquid formulation (426.0 ± 0.05 µmol Trolox equivalents (TE) and 11,336.7 ± 0.20 µmol TE in the DPPH and ORAC assays, respectively). The bioaccessibility of 6-gingerol and 6-shogaol significantly increased in the liquid form, reaching 23.44% and 11.31%, respectively, compared to ≤4% in the pure extract. These findings highlight the influence of the formulation matrix on compound release and support the use of liquid preparations to enhance the functional efficacy of ginger-derived nutraceuticals. This standardized comparative approach, using formulations derived from the same extract, offers new insights into how the delivery matrix influences the functional performance of ginger compounds, providing guidance for the development of more effective nutraceutical strategies.

## 1. Introduction

Oxidative stress is produced by an imbalance between the production of reactive oxygen species (ROS) and the ability to counteract them through antioxidant mechanisms [1,2,3]. This imbalance may be caused by insufficient antioxidant intake, the depletion of endogenous antioxidants, or an increase in ROS production [1,2]. ROS are molecules containing highly reactive oxygen; the most common examples include hydrogen peroxide (H_2_O_2_), superoxide (O_2_·) and the hydroxyl radical (OH·), the most reactive ROS species [1]. ROS are involved in essential processes in the organism, such as cellular homeostasis, gene expression, receptor activation, and signal transduction [2]. An increase in ROS production could lead to harmful effects in vital cellular structures including proteins, lipids, and nucleic acids, leading to the development of different pathological conditions that affect human health [2,3,4].

The onset of disease is closely related to cellular oxidation levels, highlighting the importance of including foods in the diet that can prevent oxidative stress [1,2,3]. Evidence from previous studies and clinical trials suggests that supplementation with phytochemicals, such as those found in *Zingiber officinale*, can decelerate cellular degradation linked to redox imbalances [5,6,7]. Ginger (*Zingiber officinale Roscoe*) is a perennial herb member of the *Zingiberaceae* family that is among the most active natural remedies due to its numerous biological properties [4]. The rhizome of *Zingiber officinale* has been used in traditional medicine for the treatment of various conditions and is composed mostly of phenolic compounds, with gingerols (6-gingerol, 8-gingerol, and 10-gingerol), shogaols (6-shogaol, 8-shogaol, and 10-shogaol), and flavonoids standing out for their antioxidant bioactivity [5,7,8,9]. These bioactive compounds exhibit their antioxidative capacity primarily through their chemical structure, characterized by multiple hydroxyl groups attached to an aromatic ring, enabling them to neutralize free radicals or chelate metal ions, thereby reducing oxidative stress [7,10].

Among the bioactive compounds present in *Zingiber officinale*, 6-shogaol and specially 6-gingerol exhibits significant in vitro antioxidant capacity [8,11]. The mechanism involved in its antioxidant capacity is related to the prevention of free radicals as well as to its ability to scavenge them [2,7]. The most recent scientific literature has shown that 6-gingerol potentiates Beclin-1 expression favoring autophagy in human endothelial cells and interferes with PI3K/AKT/mTOR pathway signaling without altering the cell cycle [2]. Different studies have shown that 6-gingerol can effectively inhibit xanthine oxidase (XO), an enzyme responsible for catalyzing the oxidation of hypoxanthine to xanthine and xanthine to uric acid, preventing the production of ROS in the last stage of metabolic degradation of purines [8]. Additionally, 6-gingerol has been reported to enhance the activity of the antioxidant enzymes superoxide dismutase (SOD) and catalase (CAT) and decrease the level of malondialdehyde (MDA), a marker of lipid peroxidation. In other research, 6-shogaol has been shown to exhibit antioxidant capacity through activation of the nuclear factor erythroid 2 (Nrf2) signaling pathway [12,13,14]. Thus, *Zingiber officinale* extract can regulate lipogenesis, fatty acid oxidation, mitochondrial dysfunction, and oxidative stress, conferring various biological properties, including anticancer, antimicrobial, anti-inflammatory, and antiallergic effects.

The effective dose of *Zingiber officinale* and the length of the administration to observe antioxidant capacity varies widely between different in vitro and in vivo studies. Daily supplementation with 1 g of ginger for up to 3 months has been shown to reduce serum nitric oxide levels and inhibit the expression of inducible nitric oxide synthase (iNOS) in immune cells, highlighting its potential role in modulating oxidative stress and inflammatory pathways [12,15]. However, the clinical translation of these findings depends not only on the activity of isolated compounds, but also on their stability, release, and absorption in real-world formulations.

Moreover, the formulation matrix plays a critical role in the protection, solubility, and biological activity of phenolic compounds such as gingerols and shogaols. Unlike specialized encapsulation technologies, conventional dosage forms like powders, capsules, or liquid solutions can modulate the stability, antioxidant capacity, and bioaccessibility of these compounds depending on the physicochemical environment they create. In this context, the liquid formulation demonstrated superior performance not only in preserving the integrity and enhancing the bioaccessibility of 6-gingerol and 6-shogaol during digestion but also in maintaining their antioxidant potential, highlighting the relevance of formulation design in maximizing the functional efficacy of ginger-derived nutraceuticals.

Despite the extensive evidence supporting the antioxidant capacity of *Zingiber officinale* and its bioactive constituents, there is limited information regarding how different pharmaceutical formulations influence its stability, solubility, and bioaccessibility under gastrointestinal conditions [16]. Most studies have focused on raw extracts or isolated compounds, overlooking the potential modifications induced by delivery systems such as capsules or liquid suspensions. These formulations may alter the release profile, protection from degradation, and ultimately the biological activity of ginger-derived compounds [17]. To address this gap, our study uses a controlled experimental design where all formulations are based on a single standardized extract of *Zingiber officinale*, allowing us to isolate the effect of the delivery matrix on antioxidant capacity and compound bioaccessibility. This approach offers a novel perspective by integrating in vitro digestion and antioxidant assessment under physiologically relevant conditions.

Therefore, it is essential to assess not only the antioxidant capacity of *Zingiber officinale* in various dosage forms, but also to understand how these formulations affect its chemical integrity and intestinal availability after digestion. By comparing different formulations, it is possible to determine the most efficient delivery system for maximizing antioxidant potential and enhancing the bioaccessibility of functional phytochemicals.

The use of nutraceuticals may serve as an adjuvant treatment for oxidative stress-related diseases, including neurodegenerative, cardiovascular, autoimmune, and inflammatory disorders [5]. In this context, the aim of this study is to evaluate the in vitro antioxidant capacity of ginger extract and capsules at different concentrations (20 mg, 40 mg, and 80 mg), as well as a liquid formulation containing *Zingiber officinale* (1000 mg of extract in 300 mL), using the oxygen radical absorbance capacity (ORAC) and 2,2-diphenyl-1-picrylhydrazyl (DPPH) methods [18,19,20], and comparing the results obtained with different solvents. Additionally, the stability and bioaccessibility of pure ginger extract and the liquid formulation will be assessed after an in vitro digestion process. Prior to these analyses, a qualitative and quantitative characterization of the ginger extract and its different pharmaceutical formulations will be conducted using high-performance liquid chromatography with diode array detection (HPLC-DAD). Therefore, these results will provide a basis for future research aimed at evaluating the in vivo bioavailability of these compounds and determining the optimal concentrations to achieve antioxidant effects in the body.

## 2. Materials and Methods

### 2.1. Chemicals and Reagents

The chemicals and solvents used in the present study were of analytical grade or higher. For sample preparation, high-performance liquid chromatography (HPLC) gradient water, acetonitrile (≥99%, CAS: 75-05-8), trifluoroacetic acid (≥99%, CAS: 76-05-1), and methanol (98%, CAS: 67-56-1) were purchased from Avantor Performance Materials (Gliwice, Poland). The standards of 6-gingerol (≥99%, CAS: 23513-14-6), 8-gingerol (≥99%, CAS: 23513-08-8), 10-gingerol (≥99%, CAS: 23513-15-7), and 6-shogaol (≥99%, CAS: 555-66-8) were obtained from Sigma-Aldrich (Madrid, Spain). 2,2′-Azobis (2-methylpropionamidine) dihydrochloride (AAPH, ≥95%, CAS: 2997-92-4), 2,2-diphenyl-1-picrylhydrazyl (DPPH, ≥95%, CAS: 1898-66-4), and 6-hydroxy-2,5,7,8-tetramethylchroman-2-carboxylic acid (Trolox, 97%, CAS: 53188-07-1) were purchased from Sigma-Aldrich (Madrid, Spain). Sodium phosphate (Na_2_HPO_4_, ≥99%, CAS: 7558-79-4), fluorescein (CAS: 518-47-8), and sodium hydroxide (NaOH, ≥95%, CAS: 1310-73-2) were obtained from PanReac AppliChem (ITW Reagents, Barcelona, Spain).

The enzymes and salts used included α-amylase (≥95%, CAS: 9000-90-2), pancreatin (≥95%, CAS: 8049-47-6), and bovine bile salts (≥90%, CAS: 8008-63-7), all acquired from Sigma-Aldrich (Madrid, Spain). The inorganic reagents used were calcium chloride dihydrate (CaCl_2_·2H_2_O, ≥99%, CAS: 10035-04-8), magnesium chloride hexahydrate (MgCl_2_·6H_2_O, ≥99%, CAS: 7791-18-6), monopotassium phosphate (KH_2_PO_4_, 99.9%, CAS: 7778-77-0), ammonium carbonate ((NH_4_)_2_CO_3_, ≥98%, CAS: 506-87-6), sodium bicarbonate (NaHCO_3_, ≥99%, CAS: 144-55-8), sodium chloride (NaCl, ≥99%, CAS: 7647-14-5), hydrochloric acid (HCl, ≥99%, CAS: 7647-01-0), and calcium dichloride (CaCl_2_, ≥99%, CAS: 10035-04-8), all purchased from PanReac AppliChem (ITW Reagents, Barcelona, Spain).

Finally, the simulated digestive fluids, including Simulated Salivary Fluid (SSF), Simulated Gastric Fluid (SGF), Fasted State Simulated Gastric Fluid (FaSSGF), 3F Powder, Simulated Intestinal Fluid (SIF), and Fasted State Simulated Intestinal Fluid (FaSSIF), were obtained from Biorelevant Ltd. (London, UK).

### 2.2. Ginger Root Extract Sample and Derived Formulations

The *Zingiber officinale* root extract used in this study was standardized to a minimum content of ≥6% total gingerols (mainly 6-, 8-, and 10-gingerol and 6-shogaol), with maltodextrin as an excipient (<8%). According to the manufacturer, 1000 mg of this dry extract approximately corresponds to between 10 and 25 g of fresh ginger rhizome, depending on the natural gingerol content in the raw material. This extract was characterized based on its physicochemical and microbiological specifications to ensure its quality and stability. It was obtained through a hydroalcoholic extraction process (ethanol/water), as described in the manufacturer’s technical sheet.

The ginger rhizomes used were cultivated in India and commercially sourced as dried raw material. Botanical identity, quality classification, and storage conditions were verified by the manufacturer prior to extraction. Based on the standardized extract, three capsule formulations were developed, containing 20 mg, 40 mg, and 80 mg of dry ginger root extract per unit, equivalent to 500 mg, 1000 mg, and 2000 mg of ginger rhizome, respectively. Additionally, the capsule formulation included excipients such as microcrystalline cellulose and magnesium stearate. Furthermore, a liquid preparation was formulated, containing 1000 mg of *Zingiber officinale* root powder extract in 300 mL of liquid formulation, composed of purified water, vegetable glycerin, potassium sorbate, and orange flavoring. The ginger root extract sample, capsules, and liquid formulation were provided by the manufacturer (Martínez Nieto S.A., Murcia, Spain). The pure ginger extract used in this study was provided as a dry powdered extract obtained through a hydroalcoholic extraction process (ethanol/water) and was standardized to a minimum of 6% gingerols.

### 2.3. HPLC-DAD Characterization of Ginger Extract and Formulations

All quantitative data are expressed as the mean ± standard deviation (SD). No statistical significance tests were applied, as the study was descriptive in nature and focused on the characterization of the different ginger formulations.

#### 2.3.1. Chromatographic Conditions

The analysis of the samples was performed by HPLC-DAD on an Agilent 1200 HPLC system equipped with a diode array detector (Agilent G1315C; Agilent Technologies, Santa Clara, CA, USA). The system included a G1312B Bin Pump, a G1329B injector, a G1316B thermostat, and a G1322A degasser. Data analysis was conducted using ChemStation software (version 1.3.1, Agilent Technologies, Santa Clara, CA, USA).

The separation of phenolic compounds was achieved using an Eclipse Plus C18 column (4.6 × 100 mm, 5 µm particle size) from Agilent Technologies (Santa Clara, CA, USA), maintained at a constant temperature of 30 °C. An isocratic elution system was employed, using a mobile phase consisting of 65% acetonitrile (mobile phase A) and 35% 0.1% trifluoroacetic acid in deionized water (mobile phase B) at a 99:1 (*v*/*v*) ratio. The separation was performed at a constant flow rate of 1.0 mL/min for 17 min, with an injection volume of 10 μL. Compound detection was carried out at 280 nm using HPLC-DAD [21].

#### 2.3.2. Preparation of Standards and Calibration Curve

The reference compounds used for the identification and quantification of the samples were 6-gingerol, 8-gingerol, 10-gingerol, and 6-shogaol. Each standard was prepared by dissolving 0.1 g of the compound in methanol, obtaining a stock solution of 1.0 mg/mL. From this stock solution, eight serial dilutions were prepared within the range of 1–50 mg/L and used for constructing the calibration curve. The solutions were stored at −20 °C in amber vials until use to prevent degradation.

#### 2.3.3. Sample Preparation

For the analysis of phenolic compounds in the ginger extract and solid pharmaceutical forms, 0.1 g of the samples was dissolved in 10 mL of methanol in a volumetric flask. The solution was subjected to ultrasonication at 40 °C for 30–40 min to facilitate the extraction of bioactive compounds, using an ultrasonic bath (Ultrasons H-D, P Selecta, Barcelona, Spain). After cooling to room temperature, the volume was adjusted to 100 mL with the mobile phase. Finally, the samples were filtered through a 0.45 µm nylon filter and stored in an amber vial until subsequent HPLC-DAD analysis.

For the analysis of the liquid formulation, a 1:2 dilution in methanol was prepared, mixed, and subjected to ultrasonication at 40 °C for 30–40 min. The solution was then centrifuged at 4000 rpm for 10 min, using a refrigerated centrifuge (Digicen 21 R, Orto Alresa, Madrid, Spain), the supernatant was filtered through a 0.45 µm nylon filter, and the resulting solution was transferred to an amber vial prior to analysis.

### 2.4. Determination of Antioxidant Capacity

The antioxidant capacity of the ginger root extract, capsules, and liquid formulation were evaluated using the DPPH and ORAC assays, which assess free radical neutralization through different mechanisms. Trolox was used as the reference standard, and the results were expressed as Trolox equivalents (TE). To ensure consistency and practical relevance, antioxidant capacity was reported as µmol TE per unit of consumption: per capsule (0.3 g total content, containing 20, 40, or 80 mg of pure ginger extract), per 0.3 g of extract, or per 300 mL of the liquid formulation. Samples were diluted as needed to fit within the linear range of the standard calibration curve.

#### 2.4.1. Evaluation of Antioxidant Activity Using the DPPH Method

The antioxidant capacity by the DPPH method was conducted following the methodology described by Arranz et al. [22] and Baliyan et al. [23], with some modifications. Extracts were obtained using three solvents with different polarities: methanol, methanol/water (50:50 *v*/*v*), and water. For this purpose, 500 mg of the sample was weighed and dissolved in 5 mL of the corresponding solvent in Falcon tubes, which were protected from light with aluminum foil. Extraction was performed by inversion mixing for 10 min, followed by ultrasonication at 40 °C for 30–40 min using an ultrasonic bath (Ultrasons H-D, P Selecta, Barcelona, Spain). The samples were then centrifuged at 4000 rpm for 15 min using a refrigerated centrifuge (Digicen 21 R, Orto Alresa, Madrid, Spain) and the supernatant was collected and filtered through a 0.45 µm nylon filter. The dilution degree of the filtered supernatant varied depending on the solvent used and the type of sample.

The DPPH free radical scavenging assay followed the method proposed by Brand-Williams et al. [24] and Bondet et al. [25] with some adaptations. This assay was performed using a Shimadzu UV-1700 UV-Vis spectrophotometer (Shimadzu Corporation, Kyoto, Japan) under dark conditions to prevent DPPH radical degradation.

Spectrophotometric readings were obtained at 517 nm, using a 1.5 mL cuvette with 900 µL of methanol as the blank. Subsequently, 100 µL of the 1.14 mM DPPH solution (0.036 g/80 mL methanol) was added, and the control absorbance (*A*_0_) was measured, ensuring an approximate value of 1.100. Then, 20 µL of the sample was incorporated, and absorbance readings were taken at 1 min intervals over 60 min. The absorbance measured at 60 min (*A*_1_) corresponded to the absorbance in the presence of the added sample. The percentage of DPPH inhibition was calculated according to the formula proposed by Yen and Duh [26] and Sirivibulkovit et al. [27]:

(1) $$\%\;DPPH\;Inhibition=\frac{\left(A_{0}-A_{1}\right)}{A_{0}}\times 100$$

Standard Trolox solutions at concentrations ranging from 0.6 to 3 mM were prepared and analyzed under the same experimental conditions described for the DPPH assay. The data obtained were fitted using linear regression, yielding a correlation coefficient (R^2^ > 0.99) and the following calibration equation:

(2) y=36.437x+7.0584

where *x* represents the Trolox concentration (mM), and *y* corresponds to the percentage of DPPH inhibition.

#### 2.4.2. Evaluation of Oxygen Radical Absorbance Capacity (ORAC)

Sample preparation was carried out following the protocol described by Ou et al. [28] and Rodríguez-Bonilla et al. [29], with minor modifications. A total of 0.3 g of each sample was weighed and dissolved in 50 mL of distilled water. The resulting solutions were vortex-mixed using a vortex mixer (Vortex-Genie 2, Scientific Industries, Bohemia, NY, USA) and then subjected to ultrasonic bath sonication at 45 °C for 90 min using an ultrasonic bath (Ultrasons H-D, P Selecta, Barcelona, Spain). The samples were centrifuged at 4000 rpm for 15 min, using a refrigerated centrifuge (Digicen 21 R, Orto Alresa, Madrid, Spain), and the supernatant was separated using a Pasteur pipette and filtered through a 0.45 µm nylon filter. Subsequently, the samples were diluted in distilled water according to their antioxidant activity to ensure that the measurements remained within the optimal range of the ORAC assay.

The ORAC assay was performed following the methodology described by Dávalos et al. [30], with modifications. The determination of antioxidant capacity was conducted using a Synergy-HT plate reader (Bio-Tek Instruments, Inc., Winooski, VT, USA) with 96-well black-walled, clear-bottom polystyrene microplates (Nalge Nunc International, Rochester, NY, USA). Fluorescence was recorded through the clear bottom of the plate using an excitation wavelength of 485/20 nm and an emission filter of 535/20 nm. A total of 100 µL of freshly prepared fluorescein solution (4 µM in 75 mM phosphate buffer, pH 7.4) was added to each well, followed by 20 µL of the sample diluted in distilled water. For the blank control, the sample was replaced with 20 µL of distilled water while maintaining the same total reaction volume. Then, 50 µL of 75 mM dipotassium phosphate buffer (pH 7.4) was added, and the plate was incubated at 37 °C for 30 min in the dark to stabilize the fluorescein. During the incubation period, a fresh AAPH solution (127 mM in phosphate buffer) was prepared. After incubation, 30 µL of the AAPH solution was added to each well to initiate the reaction, using a multichannel pipette to minimize experimental variability. Immediately afterward, the microplate was placed in the plate reader, and fluorescence measurements were recorded at regular intervals for 120 min, with automatic shaking before each reading. The antioxidant capacity of the samples was determined based on the area under the fluorescence decay curve (AUC) over time. The obtained values were compared with a Trolox standard curve within a concentration range of 6.25 to 31.25 µM.

### 2.5. Evaluation of Bioaccessibility Through In Vitro Digestion

The in vitro gastrointestinal digestion of the samples was performed using a static simulation model based on the adapted protocol by Moreno-Ortega et al. [31], employing simulated digestive fluids prepared from Biorelevant Ltd. concentrates and following the guidelines of the United States Pharmacopeia (USP 43-NF-38) when applicable.

#### 2.5.1. Preparation of Simulated Digestive Fluids

The simulated digestive fluids were prepared by adjusting their physicochemical conditions and pH to replicate the digestive environment.

SGF was prepared by combining 33.10 g of FaSSGF concentrate with 865.7 g of purified water and 0.054 g of 3F Powder. FaSSGF contains bile salts (sodium taurocholate, 0.08 mM) and phospholipids (lecithin, 0.02 mM), adjusted to pH 1.6. SIF was prepared using 33.32 g of FaSSIF concentrate, 768.9 g of distilled water, and 1.79 g of 3F Powder. FaSSIF contains bile salts (sodium taurocholate, 3.0 mM) and phospholipids (lecithin, 0.75 mM), adjusted to pH 6.5. The 3F Powder™ used in the preparation of these fluids consists of bile salts (sodium taurocholate, 15.1 mM) and phospholipids (3.69 mM) in its original form before dilution. SSF was prepared by dissolving 0.313 mL of 0.15 M MgCl_2_·6H_2_O, 9.438 mL of 0.5 M KCl, 2.313 mL of 0.5 M KH_2_PO_4_, 0.038 mL of 0.5 M (NH_4_)_2_CO_3_, and 4.250 mL of 1 M NaHCO_3_, completing the volume up to 250 mL with purified water.

All simulated digestive fluids were freshly prepared before each experiment and maintained at 37 °C under continuous agitation (100 rpm) to simulate the physiological conditions of the human digestive tract.

#### 2.5.2. In Vitro Digestion Procedure

The in vitro digestion process was performed in three phases (oral, gastric, and intestinal) under controlled temperature conditions (37 °C) using a heated stirring plate (WiseStir model SMHS-6, Witeg Labortechnik GmbH, Wertheim, Germany) in 250 mL beakers containing 5 g of each solid sample and 5 mL of the liquid formulation.

For the oral phase, 14 mL of SSF solution was added to the beakers containing the samples, along with 250 μL of human salivary α-amylase solution (1.3 mg/mL), 0.1 mL of 0.3 M CaCl_2_, and 5.65 mL of distilled water. The mixture was incubated at 37 °C with magnetic stirring at 250 rpm for 30 min. Subsequently, the gastric phase was initiated by adding 15 mL of SGF solution, along with 1.19 mL of pepsin solution (100 mg/mL in 0.1 M HCl), 0.01 mL of 0.3 M CaCl_2_, and 3.8 mL of distilled water. The mixture was incubated at 37 °C in a water bath with magnetic stirring at 400 rpm for 120 min. Next, in the intestinal phase, 22 mL of SIF, 10 mL of pancreatin solution (8 mg/mL in FaSSIF), 5 mL of bile salts (25 mg/mL in FaSSIF), 0.08 mL of 0.3 M CaCl_2_, and 9.92 mL of distilled water were added. The mixture was incubated at 37 °C with magnetic stirring at 400 rpm for 120 min.

Samples were collected before oral digestion and after the intestinal digestion phase. After completing the intestinal phase, the samples were transferred to Falcon tubes and centrifuged at 4000 rpm for 15 min. The supernatant, representing the bioaccessible fraction, was collected and stored at −20 °C for subsequent analysis by HPLC-DAD under the previously described conditions. Bioaccessibility was determined using the following equation [31,32]:

(3) $$Bioaccessibility\;(\%)=\frac{C_{f}}{C_{i}}\times 100$$

where *C_f_* represents the quantity of bioactive compounds present in the supernatant after three-phase digestion, and *C_i_* refers to the initial quantity present in the samples prior to digestion. All analyses were performed in triplicate.

This static three-phase digestion model aligns with the internationally harmonized protocols of the INFOGEST consortium [33] and adheres to recent methodological recommendations for evaluating the gastrointestinal behavior of functional foods and nutraceuticals [34]. These approaches emphasize the simulation of oral, gastric, and intestinal phases under physiologically relevant conditions to ensure the reproducibility and comparability of bioaccessibility assessments.

## 3. Results and Discussion

### 3.1. HPLC-DAD Quantification of Gingerols and Bioactive Compounds in Pharmaceutical Forms

The quantification of 6-gingerol, 8-gingerol, 10-gingerol, and 6-shogaol was performed in the various ginger-based formulations. The results are presented in Table 1.

Table 1 summarizes the quantitative results obtained for each formulation, revealing a clear and proportional relationship between the amount of extract present in the capsules and the total content of gingerols and bioactive compounds. This trend was consistent across all solid formulations and served as a reference for comparison with the powdered and liquid forms.

#### 3.1.1. Capsules and Powdered Pure Extract

In the 20 mg capsule (500 mg of ginger rhizome), 0.44 ± 0.03 mg of gingerols and 0.84 ± 0.03 mg of bioactive compounds per unit were detected. The 40 mg capsule (1000 mg of ginger rhizome) presented a proportional increase, with 0.81 ± 0.04 mg of gingerols and 1.63 ± 0.07 mg of bioactive compounds, while the 80 mg capsule (2000 mg of ginger rhizome), reached the highest values, with 1.60 ± 0.24 mg of gingerols and 3.32 ± 0.48 mg of bioactive compounds. An almost perfect correlation was observed between the amount of extract and the content of active compounds, with a coefficient of determination R^2^ = 0.999 for gingerols and R^2^ = 0.9997 for total bioactive compounds. This linearity indicates an accurate and homogeneous formulation. It was also identified that the main compounds present in all formulations were 6-gingerol and 6-shogaol, both of which are widely recognized for their potent antioxidant activity and their central role in the pharmacological properties of ginger. These compounds exert their effects primarily through radical scavenging mechanisms and the modulation of oxidative stress pathways, as demonstrated in comparative studies of ginger constituents [11]. On average, in the three capsule formulations, a total gingerol content of 0.82 ± 0.03 mg per 1 g of ginger rhizome was obtained, while the total bioactive compound content reached 1.66 ± 0.02 mg/g of ginger rhizome. These values provide a standardized reference for extrapolating the functional dose of the formulations and support the consistency of the conversion ratio between the extract and the rhizome.

For the pure extract, the results were expressed in percentage, obtaining a content of 1.84 ± 0.01% of gingerols and 4.02 ± 0.01% of bioactive compounds. These values confirm the standardization of the extract and its potential as a concentrated source of active principles. On a per-gram-of-fresh-rhizome equivalent basis, the pure extract specifications correspond to 1.84 ± 0.01 mg total gingerols and 4.02 ± 0.01 mg total bioactives. In contrast, the three capsule strengths average 0.82 ± 0.03 mg total gingerols and 1.66 ± 0.02 mg total bioactives per gram of rhizome. These capsule values represent approximately 45% of the extract’s gingerol level and 41% of its total bioactive content, with the difference reflecting dilution by microcrystalline excipients rather than the loss or degradation of the active compounds during encapsulation.

Additionally, regarding the absolute content, the relative proportion of these compounds with respect to the total weight of the capsules was calculated. On average, considering the three formulations, the content of gingerols was approximately 0.021 mg/mg (2.1%) and that of bioactive compounds was 0.041 mg/mg (4.1%). These values are consistent with those obtained for the pure extract, which showed a content of 1.84 ± 0.01% gingerols and 4.02 ± 0.01% bioactive compounds, suggesting that the encapsulated formulation adequately preserves the proportion of active principles of the original extract.

This information is significant to evaluate the potential efficacy of the formulations, as it allows estimation of the number of capsules needed to reach an effective dose and ensures the consistency of the product in terms of the quality and concentration of bioactive compounds.

The results obtained in this study are consistent with those reported by Pawar et al. [35], who used HPLC to quantify the 6-gingerol content in different types of ginger cultivars. In their investigation, the 6-gingerol content was significantly higher in conventionally grown rhizomes (0.165%, equivalent to 1.65 mg per gram of dried rhizome) compared to those obtained from callus (0.056%) and micropropagated rhizomes (0.078%). This difference was attributed to the lower capacity of in vitro cultures to synthesize secondary metabolites, such as gingerols, due to cell dedifferentiation. Expressing these concentrations relative to the rhizome equivalent, rather than per capsule, provides a standardized and extrapolatable metric that facilitates meaningful comparisons across studies and natural sources. This approach accounts for variability in capsule size and formulation, offering a more robust basis for evaluating the functional potency of ginger-derived products. The quantified values indicate a high degree of extract standardization and are consistent with, or slightly below, the concentrations typically reported for conventionally dried ginger rhizomes, which range from 1.65 mg/g to 4.4 mg/g of 6-gingerol depending on the cultivar and processing method [36]. These findings support the efficacy of the purified extract in delivering reproducible and physiologically relevant doses of bioactive compounds in encapsulated formats.

Likewise, the pure extract used in this study showed a content of 1.84% gingerols, which represents a concentration more than ten times higher than that reported by Pawar et al. [35] for conventional rhizomes. This difference can be explained by the use of purified and standardized extracts in our formulation, as opposed to the crude extracts used in previous studies. In addition, our 80 mg capsules (2000 mg of ginger rhizome), also contained 1.65 mg/g rhizome of total bioactive compounds, which represent a considerably higher dose than that found in fresh or in vitro-grown rhizomes.

These findings are also in agreement with those reported by Cha et al. [37], who optimized the extraction process of functional compounds from ginger and achieved concentrations of up to 39.55 mg/g of 6-gingerol and 2.44 mg/g of 6-shogaol by 70% ethanol extraction and HPLC analysis. In a complementary manner, Johnson et al. [38], reported average concentrations of 0.44% 6-gingerol and 0.144% 6-shogaol in conventional dried ginger, values that are in line with those of Pawar et al. and reinforce the significant difference with respect to the purified extracts used in our study [35].

#### 3.1.2. Liquid Formulation

In addition to the solid formulations, a liquid preparation containing 1000 mg of *Zingiber officinale* root powder pure extract in 300 mL was also analyzed. This extract was standardized to a gingerol content of ≥6% and was equivalent to 1000 mg of ginger rhizome. The HPLC-DAD analysis revealed a total gingerol content of 20.50 ± 0.30 mg per 300 mL and a total of 45.55 ± 0.30 mg of bioactive compounds. These values can also be expressed in terms of concentration, which corresponds to 68.33 mg/L of gingerols and 151.83 mg/L of total bioactive compounds.

To express these results per gram of fresh rhizome, the manufacturer’s specified equivalence range of 10–25 g fresh ginger rhizome per 1 g extract was adopted and the midpoint (17.5 g/g) selected for all conversions. Under this assumption, the liquid formulation yields 1.17 ± 0.02 mg total gingerols/g of ginger rhizome and 2.60 ± 0.02 mg total bioactive compounds/g of ginger rhizome. Across the full 10–25 g range, these values may vary from 0.82–2.05 mg/g for total gingerols and 1.82–4.56 mg/g for total bioactives. Compared with the capsule averages of 0.824 ± 0.029 mg total gingerols/g rhizome and 1.657 ± 0.022 mg total bioactive/g rhizome, the liquid delivers a 1.42-fold (42%) increase in total gingerols and a 1.57-fold (57%) increase in total bioactives, underscoring its superior active-compound density and the likely enhanced bioefficacy of the solubilized, non-compressed matrix. These 1.4- and 1.57-fold increases in active compounds highlight the higher density of active ingredients and the likely improvement in the bioefficacy of the liquid formulation. This is attributed to its completely solubilized matrix and the absence of compression or encapsulation processes that can delay the release of the compounds. Consequently, the liquid form could offer a more efficient delivery system for ginger’s bioactive compounds, promoting faster absorption and greater systemic availability [39,40].

Compared with the pure extract standardized to 1.84 ± 0.01 mg total gingerols and 4.02 ± 0.01 mg total bioactive per gram of ginger rhizome equivalent, the liquid formulation delivers 1.17 ± 0.02 mg and 2.60 ± 0.02 mg, respectively. This represents a 0.64-fold change (36% lower) for gingerols and a 0.65-fold change (35% lower) for total bioactives. These modest differences fall within analytical and formulation variability, confirming that the solubilized liquid matrix preserves most of the extract’s active-compound profile while offering the added benefits of enhanced solubility and application versatility.

The results obtained for the liquid formulation in this study, 68.33 mg/L of gingerols and 151.83 mg/L of total bioactive compounds, are notably higher than those typically reported in commercial or experimental ginger beverages. For instance, González-González et al. [41] quantified gingerols and shogaols in ginger extracts obtained via microwave-assisted extraction and reported maximum concentrations of approximately 4.4 mg/g (4400 mg/kg) for 6-gingerol and 1.44 mg/g (1440 mg/kg) for 6-shogaol in dried ginger samples. While these values refer to raw material, they highlight the challenge of achieving high concentrations in final beverage formulations.

In comparison, most commercial ginger drinks or infusions contain significantly lower levels of active compounds. For example, Ayustaningwarno et al. [42] reported that traditional ginger beverages prepared by decoction typically yield 6-gingerol concentrations ranging from 2.5 to 12.0 mg/L, depending on the extraction time and ginger-to-water ratio of 2. Similarly, Tohma et al. [43], found that cold-pressed ginger juices and infusions rarely exceed 15–20 mg/L of total gingerols.

The liquid formulation analyzed in this study, standardized to a gingerol content of ≥6%, delivered more than three times the gingerol concentration of the most potent traditional preparations, and up to ten times that of typical commercial beverages. This enhanced potency is likely due to the use of a purified extract and optimized formulation matrix, which improves the solubility and stability of the phenolic compounds.

### 3.2. In Vitro Antioxidant Capacity

#### 3.2.1. Antioxidant Capacity Determined by the DPPH Method

The antioxidant capacity of the different ginger formulations, evaluated using the DPPH method, exhibited a marked dependence on the type of solvent used and the pharmaceutical form analyzed. As shown in Table 2, methanol was the most effective solvent for extracting antioxidant compounds, followed by the methanol/water mixture (50:50), whereas water showed a very limited capacity across all samples.

This difference can be attributed to the polarity of the solvents and the chemical nature of the phytochemicals present in ginger, such as gingerols, shogaols, paradols, and zingerone, which are more soluble in organic or hydroalcoholic media [44]. Studies such as those by Ezez and Tefera [45] and Jorge-Montalvo et al. [46] have shown that methanolic extracts have a greater antioxidant activity than aqueous extracts in correlation with a higher total phenolic content, due to their ability to penetrate cells and solubilize polar and nonpolar metabolites.

The hydroalcoholic extracts showed moderate antioxidant capacity, reinforcing the hypothesis that the polarity of the extraction system affects the efficiency of bioactive compound recovery. In this regard, Numan et al. concluded that methanol/water mixtures offer a favorable balance between the solubility and recovery of phenolic compounds with a high reducing capacity [47]. This pattern was also supported by an institutional study conducted at María Auxiliadora University (Lima, Peru), where it was observed that hydroalcoholic extracts of *Zingiber Officinale* had a significantly higher antioxidant capacity than aqueous extracts, in direct correlation with a higher content of total phenolic compounds [48].

In addition to the influence of the solvent, the results could be explained by the qualitative composition of the phenolic compounds present in the extracts. Although the total amount of phenolic compounds is usually correlated with antioxidant activity, recent studies have shown that not all phenols are equally effective in neutralizing the DPPH radical. For example, the work of Dugasani et al. showed that 6-shogaol had a greater antioxidant capacity than gingerols (6-, 8-, and 10-gingerol), which is attributed to the presence of a highly reactive α, β-unsaturated carbonyl group [11]. However, other studies also highlight the high antioxidant potential of 6-gingerol against the DPPH radical, especially due to its natural abundance in the rhizome and its ability to stabilize free electrons. Taken together, these findings suggest that both the relative proportion and the possible synergy between 6-gingerol and 6-shogaol contribute to the total antioxidant efficacy of the extract.

Furthermore, the results obtained in this study are comparable to those reported in the literature. Stoilova et al. used methanol as a solvent and reported the DPPH values between 40 and 60 µmol TE per 0.3 g of extract, a range that coincides with that observed in our pure extract (54.90 ± 0.40 µmol TE), which supports the validity of our results, and the effectiveness of the standardized extract evaluated [42,49].

From a formulation perspective, the pharmaceutical form had a considerable impact on the results. The capsules showed dose-dependent behavior, increasing in antioxidant activity as the amount of extract increased. However, even in these formulations, extraction with water proved to be ineffective, suggesting that traditional ginger infusions may have a limited antioxidant capacity compared to more concentrated formulations or those formulated with suitable solvents.

The pure extract had a higher antioxidant capacity than the capsules, which is attributable to its high concentration of active ingredients (≥6% gingerols) and the absence of excipients that could interfere with their release. However, it was the liquid formulation that showed the highest antioxidant capacity. Despite its lower concentration per milliliter, the total volume of 300 mL reached 426 ± 0.05 µmol TE. This performance may be due to the better dispersion of phenolic compounds, the greater availability of the bioactive fraction, and the potential use of technologies that promote the solubilization and stability of antioxidants, as has been observed in recent studies [19,50,51].

This finding reinforces the importance of considering the actual consumption dose as a relevant parameter in the functional evaluation of nutraceutical formulations, a perspective also noted by other authors when comparing solid and liquid products in bioaccessibility and bioavailability studies [19,51]. Although many studies express DPPH results as the IC_50_, in this work we chose to report the results as µmol TE per unit of consumption, given the specific dilution used. The calculation of IC_50_ is proposed as a future line of study.

#### 3.2.2. Antioxidant Capacity Determined Using the ORAC Method

The antioxidant capacity evaluated by the ORAC assay revealed significant differences among the ginger-based formulations, with values ranging from 15.65 ± 0.37 µmol TE for the 20 mg capsule to 11,336.7 ± 0.20 µmol TE for the liquid formulation (Table 3). This ascending trend, correlated with extract content, aligns with the findings of Ghasemzadeh et al., who reported in different investigations a direct association between gingerol and shogaol concentrations and peroxyl radical scavenging efficiency as measured by the ORAC method [52,53].

The pure extract (0.3 g) yielded a value of 791.3 ± 1.20 µmol TE, highlighting its high antioxidant density. Comparable results have been reported by Hoferl et al., who documented ORAC values exceeding 700 µmol TE/g in concentrated Ecuadorian ginger extracts [54]. Likewise, Grabsk et al. observed values between 810 and 840 µmol TE/g in ginger extracts incorporated into lipid matrices, depending on the extraction protocol and experimental conditions [55].

Of particular interest, the exceptionally high ORAC value observed in the liquid formulation (11,336.7 ± 0.20 µmol TE/300 mL) falls within the range previously reported by Taskeen et al., who found values between 9000 and 13,000 µmol TE in standardized ginger infusions prepared under optimized temperature and time parameters [56]. These data underscore the relevance of not only the concentration of bioactives, but also the pharmaceutical form and volume ingested—critical factors that modulate the functional antioxidant efficacy of the final product.

In terms of formulation performance, the capsules displayed a clear dose-dependent response but yielded moderate absolute ORAC values. This may be attributed to both the lower extract quantity per unit dose and the solid matrix, which may hinder the immediate release of active compounds in aqueous environments.

Although not commonly used as a direct consumption form, the pure extract showed superior functional density, attributed to its high gingerol and shogaol content and the absence of interfering excipients. However, the liquid formulation exhibited the highest antioxidant performance overall. This effect may result from the improved dispersion and solubility of phenolic compounds, the greater homogeneity of the system, and the possible application of formulation technologies aimed at enhancing the stability and bioavailability of antioxidants [7].

Collectively, these findings reinforce prior evidence and support the hypothesis that the antioxidant activity of ginger-based preparations is governed by three interrelated factors: the pharmaceutical form, the concentration of active constituents, and the physicochemical characteristics of the delivery matrix. These variables will be further contextualized in the comparative analysis presented in the following section.

#### 3.2.3. Comparative Evaluation of Antioxidant Capacity Using DPPH and ORAC Assays

The DPPH and ORAC methods, although both intended to assess the in vitro antioxidant capacity, operate through different radical-based mechanisms that influence assay sensitivity and the class of compounds detected. DPPH quantifies the transfer of electrons or hydrogen atoms to a stable radical in solution, whereas ORAC measures the capacity to neutralize peroxyl radicals generated by AAPH in an aqueous medium, thereby better simulating physiological oxidative stress conditions.

This methodological divergence explains the higher antioxidant values obtained via ORAC across all formulations, particularly in the liquid formulation (11,336.7 ± 0.20 µmol TE), as compared to those determined using DPPH (426.0 ± 0.05 µmol TE). Nevertheless, both assays demonstrated a consistent trend in relative performance: the liquid formulation exhibited the highest antioxidant capacity, followed by the pure extract, and then the capsules, reflecting a dose-dependent behavior.

These differences are not attributable to variations in the samples themselves, but rather to the distinct affinities of bioactive compounds for the radicals involved and the physicochemical properties of the reaction media. ORAC is particularly sensitive to water-soluble phenolic compounds such as 6-shogaol, whose α, β-unsaturated carbonyl structure confers high reactivity toward peroxyl radicals.

While both assays present inherent limitations, as they are chemical in nature and do not account for physiological processes such as bioavailability or metabolism, their combined application provides a broader and more nuanced perspective on the functional antioxidant potential of the evaluated formulations. These discrepancies are explained not only by the underlying assay mechanisms, but also by the interactions between ginger phenolics and the surrounding medium, as reported in previous studies on the stability and release of *Zingiber officinale* bioactives in complex liquid matrices [57].

**Figure 1:**
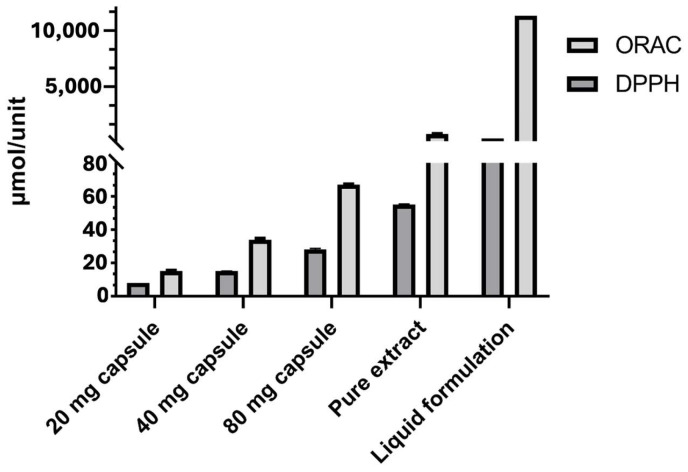
Comparison of antioxidant capacity determined by DPPH (using methanol as the solvent) and ORAC assays in different formulations of *Zingiber officinale*. Results are expressed as µmol TE per unit of consumption: per capsule, per 0.3 g of pure extract, or per 300 mL of liquid formulation. Error bars represent the standard deviation.

The comparative overview provided by DPPH and ORAC (Figure 1) underscores the critical importance of factors such as the pharmaceutical form, the effective concentration of active compounds, and the dissolution matrix in designing *Zingiber officinale* formulations with clinically relevant antioxidant properties.

### 3.3. Bioaccessibility After In Vitro Digestion

The bioaccessibility of the main phenolic compounds in ginger was evaluated using an in vitro gastrointestinal digestion model to determine the proportion of each compound that reaches the intestinal phase under simulated human digestion conditions. Two matrices were compared: a pure ginger root extract and a liquid formulation derived from it. The results, presented in Table 4, revealed a differential release pattern of the analyzed compounds (6-gingerol, 8-gingerol, 10-gingerol, and 6-shogaol) during the intestinal phase. In the pure extract, all four compounds were detected, although their bioaccessibility percentages were low: 3.15% for 6-gingerol, 3.88% for 8-gingerol, 1.24% for 10-gingerol, and 1.92% for 6-shogaol. In contrast, in the liquid formulation, only 6-gingerol (23.44%) and 6-shogaol (11.31%) were quantifiable, with no detection of the longer-chain gingerols.

This pattern may be partly explained by the higher relative abundance of 6-gingerol and 6-shogaol in both matrices, as well as by their physicochemical characteristics. Their lower alkyl chain length and molecular weight enhance solubility in the digestive environment and promote incorporation into intestinal micelles. In contrast, the longer-chain gingerols (8- and 10-gingerol) exhibit lower solubility and may be more susceptible to chemical transformation during digestion, limiting their recovery.

The liquid formulation exhibited a more efficient release of the main compounds, likely due to its aqueous matrix and colloidal structure, which support the solubilization and stabilization of lipophilic compounds during gastrointestinal transit. Such systems have been widely recognized in the literature for their ability to enhance the intestinal release of active compounds. Arcusa et al. emphasized that gingerols require colloidal carriers or emulsifiers for effective micellar incorporation [2], while other authors have shown that liquid formulations significantly improve the bioavailability of natural antioxidants by facilitating their dispersion and persistence in the digestive milieu [3].

Of particular interest is the study by Zagórska et al., who examined how different dietary patterns influence the bioavailability of ginger polyphenols using in vitro digestion. They found that a high-fiber diet substantially increased the bioavailability of 6-gingerol (up to 33.3%) compared to a standard diet (21.3%) [58]. In our study, the liquid formulation achieved a value of 23.44% for this compound. This suggests that well-designed liquid delivery systems may replicate, at least in part, the functional benefits of a high-fiber diet without requiring dietary changes from the consumer.

Moreover, previous studies by Moreno-Ortega et al. [31] and Contreras-López et al. [59] reported 6-gingerol bioaccessibility values ranging from 4% to 25%, depending on the matrix and experimental conditions. Our findings fall within this range, supporting both the validity of the in vitro model and the effectiveness of the liquid formulation used.

From a physiological standpoint, Szymczak et al. compiled clinical data showing that peak plasma concentrations of gingerols and shogaols occur between 1 and 3 h after oral ingestion, although with rapid hepatic metabolism and a short half-life limit systemic exposure [60]. Similarly, Songvut et al. demonstrated that 6-gingerol and 6-shogaol, when administered as isolated compounds, exhibit rapid but limited intestinal absorption (<2%) in animal models due to biotransformation and excretion [61]. These findings underscore the strategic relevance of liquid or colloidal formulations as advanced delivery systems that enhance the absorbable fraction of bioactive compounds. As highlighted by Shaker et al., the pharmacokinetic properties of these compounds, along with their matrix interactions and mode of administration, are key determinants of their clinical efficacy [62]. Comparable results have also been observed in studies with other lipophilic compounds, such as lutein, where colloidal formulations significantly increased bioaccessibility and bioavailability, reinforcing the potential of this strategy for natural compounds with low aqueous solubility [63].

## 4. Conclusions

This study demonstrates that the antioxidant capacity and bioaccessibility of ginger-derived compounds are significantly influenced by the pharmaceutical form. Among the formulations tested, the liquid form exhibited superior antioxidant activity—particularly in the ORAC assay—and greater bioaccessibility of 6-gingerol and 6-shogaol following in vitro digestion. These two compounds, which are the primary contributors to ginger’s functional activity, showed markedly enhanced release in the liquid matrix compared to the pure extract.

These findings have strong applied relevance, positioning liquid formulations as a more effective alternative for optimizing the release, solubility, and stability of ginger phenolics under physiological conditions. From a scientific perspective, the study provides valuable evidence on the role of formulation matrix in the functionality of nutraceuticals, contributing to the rational design of products aimed at mitigating oxidative stress and promoting preventive health.

Future research should focus on in vivo bioavailability studies to confirm the systemic absorption of these compounds, as well as on the clinical evaluation of their long-term efficacy. This approach will help to advance the scientific validation and therapeutic application of *Zingiber officinale*-based products in the context of oxidative stress-related diseases.

## Figures and Tables

**Table 1:** Total content of gingerols and bioactive compounds in ginger capsules (20 mg, 40 mg, and 80 mg dry extract), powdered pure extract (6%), and liquid formulation (300 mL). Values are expressed in mg per capsule, in percentage (%) mg/300 mL, and equivalents in mg/g rhizome.

Sample	Bioactive Compound	Result	Total Gingerols		Total Active Compounds	
			Eq.		Eq.	
20 mg capsule (500 mg of ginger rhizome)	6-Gingerol	0.26 ± 0.01 mg/cap	0.44 ± 0.03 mg/cap	0.87 ± 0.06 mg/g rhizome	0.84 ± 0.03 mg/cap	1.69 ± 0.07 mg/g rhizome
	8-Gingerol	0.04 ± 0.01 mg/cap				
	10-Gingerol	0.13 ± 0.02 mg/cap				
	6-Shogaol	0.41 ± 0.02 mg/cap				
40 mg capsule (1000 mg of ginger rhizome)	6-Gingerol	0.50 ± 0.02 mg/cap	0.81 ± 0.04 mg/cap	0.81 ± 0.04 mg/g rhizome	1.63 ± 0.07 mg/cap	1.63 ± 0.07 mg/g rhizome
	8-Gingerol	ND				
	10-Gingerol	0.31 ± 0.02 mg/cap				
	6-Shogaol	0.82 ± 0.04 mg/cap				
80 mg capsule (2000 mg of Ginger rhizome)	6-Gingerol	1.04 ± 0.14 mg/cap	1.60 ± 0.24 mg/cap	0.80 ± 0.12 mg/g rhizome	3.32 ± 0.48 mg/cap	1.66 ± 0.24 mg/g rhizome
	8-Gingerol	0.10 ± 0.04 mg/cap				
	10-Gingerol	0.47 ± 0.06 mg/cap				
	6-Shogaol	1.72 ± 0.24 mg/cap				
Pure extract (6% purity)	6-Gingerol	1.187 ± 0.006%	1.84 ± 0.01%	1.84 ± 0.01 mg/g rhizome	4.02 ± 0.01%	4.02 ± 0.01 mg/g rhizome
	8-Gingerol	0.215 ± 0.005%				
	10-Gingerol	0.442 ± 0.013%				
	6-Shogaol	2.173 ± 0.006%				
Liquid formulation 300 mL (1000 mg of *Zingiber officinale*)	6-Gingerol	13.57 ± 0.13 mg/300 mL	20.50 ± 0.30 mg/300 mL	1.17 ± 0.02 mg/g rhizome	45.55 ± 0.30 mg/300 mL	2.60 ± 0.02 mg/g rhizome
	8-Gingerol	2.56 ± 0.10 mg/300 mL				
	10-Gingerol	4.37 ± 0.39 mg/300 mL				
	6-Shogaol	25.05 ± 0.49 mg/300 mL				

Cap: capsule; Eq: equivalent; ND: not detected under the assay conditions. Data are expressed as the mean ± standard deviation; mg/g rhizome expresses the amount of compound per gram of fresh ginger rhizome equivalent. All calculations assume 1 g dry extract = 17.5 g fresh rhizome.

**Table 2:** Antioxidant capacity of *Zingiber officinale* formulations in different solvents, determined using the DPPH method. Values are expressed as µmol TE per unit of consumption: per capsule, per 0.3 g of pure extract, or per 300 mL of liquid formulation.

Sample	Methanol	Water	Methanol/Water
20 mg capsule (500 mg of ginger rhizome)	8.28 ± 0.03	0.90 ± 0.01	6.61 ± 0.01
40 mg capsule (1000 mg of ginger rhizome)	15.30 ± 0.20	1.86 ± 0.01	12.09 ± 0.35
80 mg capsule (2000 mg of Ginger rhizome)	28.20 ± 0.80	2.71 ± 0.02	21.42 ± 0.15
Pure extract (0.3 g)	54.90 ± 0.40	2.44 ± 0.02	39.21 ± 0.08
Liquid formulation	426.00 ± 0.05	ND	314.00 ± 0.04

ND: not detected under the assay conditions. Data are expressed as the mean ± standard deviation.

**Table 3:** Antioxidant capacity determined using the ORAC method in different formulations of *Zingiber officinale*. Values expressed as µmol TE per unit of consumption: per capsule, per 0.3 g of pure extract, or per 300 mL of liquid formulation.

Sample	Antioxidant Capacity
20 mg capsule (500 mg of ginger rhizome)	15.65 ± 0.37
40 mg capsule (1000 mg of ginger rhizome)	33.8 ± 0.57
80 mg capsule (2000 mg of ginger rhizome)	66.80 ± 0.11
Pure extract (0.3g)	791.3 ± 1.20
Liquid formulation	11,336.7 ± 0.20

Data are expressed as mean ± standard deviation.

**Table 4:** Content and intestinal bioaccessibility percentage of the main bioactive compounds from *Zingiber officinale* following in vitro digestion in the pure extract and the liquid formulation.

Sample	Bioactive Compound	C_i_ (mg)	C_f_ (mg)	Bioaccessibility (%)
Pure extract	6-gingerol	59.36 ± 0.29	1.87 ± 0.29	3.15 ± 0.49
	8-gingerol	10.74 ± 0.23	0.42 ± 0.17	3.88 ± 1.60
	10-gingerol	22.08 ± 0.65	0.27 ± 0.06	1.24 ± 0.30
	6-shogaol	108.64 ± 0.28	2.08 ± 0.54	1.92 ± 0.49
Liquid Formulation	6-gingerol	0.23 ± 0.01	0.05 ± 0.03	23.44 ± 1.24
	8-gingerol	0.04 ± 0.01	ND	ND
	10-gingerol	0.07 ± 0.01	ND	ND
	6-shogaol	0.42 ± 0.01	0.05 ± 0.01	11.31 ± 1.24

ND: not detected under the assay conditions; C_f_ represents the amount of bioactive compound (mg) in the supernatant after three-phase digestion, and C_i_ refers to the initial amount (mg) prior to digestion. Results are expressed as the mean ± standard deviation.

## Data Availability

The original contributions presented in this study are included in the article. Further inquiries can be directed to the corresponding author.

## References

[1] Frijhoff J., Winyard P.G., Zarkovic N., Davies S.S., Stocker R., Cheng D., Knight A.R., Taylor E.L., Oettrich J., Ruskovska T. (2015). Clinical Relevance of Biomarkers of Oxidative Stress. Antioxid. Redox Signal..

[2] Arcusa R., Villaño D., Marhuenda J., Cano M., Cerdà B., Zafrilla P. (2022). Potential Role of Ginger (*Zingiber officinale* Roscoe) in the Prevention of Neurodegenerative Diseases. Front. Nutr..

[3] Kadam S.N., Abhonkar R.S., Ahire P.S. (2022). The Review on Medicinal Uses of Ginger. IJFMR Int. J. Multidiscip. Res..

[4] Syafitri D.M., Levita J., Mutakin M., Diantini A. (2018). A Review: Is Ginger (*Zingiber officinale* Var. Roscoe) Potential for Future Phytomedicine?. Indones. J. Appl. Sci..

[5] Shaukat M.N., Nazir A., Fallico B. (2023). Ginger Bioactives: A Comprehensive Review of Health Benefits and Potential Food Applications. Antioxidants.

[6] Cerdá B., Marhuenda J., Arcusa R., Villaño D., Ballester P., Zafrilla P. (2022). El Jengibre En La Prevención de Enfermedades Cardiovasculares. Jengibre: Propiedades Funcionales y Usos Terapéuticos.

[7] Mao Q.Q., Xu X.Y., Cao S.Y., Gan R.Y., Corke H., Beta T., Li H.B. (2019). Bioactive Compounds and Bioactivities of Ginger (*Zingiber officinale* Roscoe). Foods.

[8] Ahmed S.H.H., Gonda T., Agbadua O.G., Girst G., Berkecz R., Kúsz N., Tsai M.C., Wu C.C., Balogh G.T., Hunyadi A. (2023). Preparation and Evaluation of 6-Gingerol Derivatives as Novel Antioxidants and Antiplatelet Agents. Antioxidants.

[9] Ezzat S.M., Ezzat M.I., Okba M.M., Menze E.T., Abdel-Naim A.B. (2018). The Hidden Mechanism beyond Ginger (*Zingiber officinale* Rosc.) Potent in Vivo and in Vitro Anti-Inflammatory Activity. J. Ethnopharmacol..

[10] Alolga R.N., Wang F., Zhang X., Li J., Tran L.S.P., Yin X. (2022). Bioactive Compounds from the *Zingiberaceae* Family with Known Antioxidant Activities for Possible Therapeutic Uses. Antioxidants.

[11] Dugasani S., Pichika M.R., Nadarajah V.D., Balijepalli M.K., Tandra S., Korlakunta J.N. (2010). Comparative Antioxidant and Anti-Inflammatory Effects of [6]-Gingerol,[8]-Gingerol,[10]-Gingerol and [6]-Shogaol. J. Ethnopharmacol..

[12] Naderi Z., Mozaffari-Khosravi H., Dehghan A., Nadjarzadeh A., Huseini H.F. (2016). Effect of Ginger Powder Supplementation on Nitric Oxide and C-Reactive Protein in Elderly Knee Osteoarthritis Patients: A 12-Week Double-Blind Randomized Placebo-Controlled Clinical Trial. J. Tradit. Complement. Med..

[13] Park G., Oh D.-S., Lee M.G., Lee C.E., Kim Y. (2016). 6-Shogaol, an Active Compound of Ginger, Alleviates Allergic Dermatitis-like Skin Lesions via Cytokine Inhibition by Activating the Nrf2 Pathway. Toxicol. Appl. Pharmacol..

[14] Peng S., Yao J., Liu Y., Duan D., Zhang X., Fang J. (2015). Activation of Nrf2 Target Enzymes Conferring Protection against Oxidative Stress in PC12 Cells by Ginger Principal Constituent 6-Shogaol. Food Funct..

[15] Crichton M., Davidson A.R., Innerarity C., Marx W., Lohning A., Isenring E., Marshall S. (2022). Orally Consumed Ginger and Human Health: An Umbrella Review. Am. J. Clin. Nutr..

[16] Mukkavilli R., Yang C., Tanwar R.S., Ghareeb A., Luthra L., Aneja R. (2017). Absorption, Metabolic Stability, and Pharmacokinetics of Ginger Phytochemicals. Molecules.

[17] Mukkavilli R., Yang C., Tanwar R.S., Saxena R., Gundala S.R., Zhang Y., Ghareeb A., Floyd S.D., Vangala S., Kuo W.-W. (2018). Pharmacokinetic-Pharmacodynamic Correlations in the Development of Ginger Extract as an Anticancer Agent. Sci. Rep..

[18] Jiménez-Pulido I.J., Martín-Diana A.B., Luis D.D., Rico D. (2024). Comparative Bioaccesibility Study of Cereal-Based Nutraceutical Ingredients Using INFOGEST Static, Semi-Dynamic and Dynamic In Vitro Gastrointestinal Digestion. Antioxidants.

[19] Barbari R., Bruggink V., Hofstetter R.K., Tupini C., Fagnani S., Baldini E., Durini E., Lampronti I., Vertuani S., Baldisserotto A. (2024). Synthesis and Biological Activity Assessment of 2-Styrylbenzothiazoles as Potential Multifunctional Therapeutic Agents. Antioxidants.

[20] Nam D.G., Kim M., Choi A.J., Choe J.S. (2024). Health Benefits of Antioxidant Bioactive Compounds in Ginger (*Zingiber officinale*) Leaves by Network Pharmacology Analysis Combined with Experimental Validation. Antioxidants.

[21] European Directorate for the Quality of Medicines & HealthCare (EDQM) (2020). European Pharmacopoeia.

[22] Arranz S., Cert R., Pérez-Jiménez J., Cert A., Saura-Calixto F. (2008). Comparison between Free Radical Scavenging Capacity and Oxidative Stability of Nut Oils. Food Chem..

[23] Baliyan S., Mukherjee R., Priyadarshini A., Vibhuti A., Gupta A., Pandey R.P., Chang C.-M. (2022). Determination of Antioxidants by DPPH Radical Scavenging Activity and Quantitative Phytochemical Analysis of *Ficus religiosa*. Molecules.

[24] Brand-Williams W., Cuvelier M.E., Berset C. (1995). Use of a Free Radical Method to Evaluate Antioxidant Activity. LWT Food Sci. Technol..

[25] Bondet V., Brand-Williams W., Berset C. (1997). Cinética y Mecanismos de La Actividad Antioxidante Mediante El Método de Radicales Libres. LWT Food Sci. Technol..

[26] Yen G.-C., Duh P.-D. (1995). Antioxidant Activity of Methanolic Extracts of Peanut Hulls from Various Cultivars. J. Am. Oil Chem. Soc..

[27] Sirivibulkovit K., Nouanthavong S., Sameenoi Y. (2018). Paper-Based DPPH Assay for Antioxidant Activity Analysis. Anal. Sci..

[28] Ou B., Huang D., Hampsch-Woodill M., Flanagan J.A., Deemer E.K. (2002). Analysis of Antioxidant Activities of Common Vegetables Employing Oxygen Radical Absorbance Capacity (ORAC) and Ferric Reducing Antioxidant Power (FRAP) Assays:  A Comparative Study. J. Agric. Food Chem..

[29] Rodríguez-Bonilla P., Gandía-Herrero F., Matencio A., García-Carmona F., López-Nicolás J.M. (2017). Comparative Study of the Antioxidant Capacity of Four Stilbenes Using ORAC, ABTS+, and FRAP Techniques. Food Anal. Methods.

[30] Dávalos A., Gómez-Cordovés C., Bartolomé B. (2004). Extending Applicability of the Oxygen Radical Absorbance Capacity (ORAC-Fluorescein) Assay. J. Agric. Food Chem..

[31] Moreno-Ortega A., Pereira-Caro G., Ordóñez J.L., Moreno-Rojas R., Ortíz-Somovilla V., Moreno-Rojas J.M. (2020). Bioaccessibility of Bioactive Compounds of ‘Fresh Garlic’ and ‘Black Garlic’ Through In Vitro Gastrointestinal Digestion. Foods.

[32] Gomes T.M., Toaldo I.M., da Silva Haas I.C., Burin V.M., Caliari V., Luna A.S., de Gois J.S., Bordignon-Luiz M.T. (2019). Differential Contribution of Grape Peel, Pulp, and Seed to Bioaccessibility of Micronutrients and Major Polyphenolic Compounds of Red and White Grapes through Simulated Human Digestion. J. Funct. Foods.

[33] Egger L., Ménard O., Delgado-Andrade C., Alvito P., Assunção R., Balance S., Barberá R., Brodkorb A., Cattenoz T., Clemente A. (2016). El Método Armonizado de Digestión in Vitro INFOGEST: Del Conocimiento a La Acción. Food Res. Int..

[34] Dávila León R., González-Vázquez M., Lima-Villegas K.E., Mora-Escobedo R., Calderón-Domínguez G. (2023). In Vitro Gastrointestinal Digestion Methods of Carbohydrate-rich Foods. Food Sci. Nutr..

[35] Pawar N.V., Pai S.R., Nimbalkar M.S., Dixit G.B. (2015). RP-HPLC Analysis of Phenolic Antioxidant Compound 6-Gingerol from in Vitro Cultures of *Zingiber officinale* Roscoe. Plant Sci Today.

[36] Semwal R.B., Semwal D.K., Combrinck S., Viljoen A.M. (2015). Gingerols and Shogaols: Important Nutraceutical Principles from Ginger. Phytochemistry.

[37] Cha J., Kim C.-T., Cho Y.-J. (2020). Optimizing Extraction Conditions for Functional Compounds from Ginger (*Zingiber officinale* Roscoe) Using Response Surface Methodology. Food Sci. Biotechnol..

[38] Johnson J.B., Mani J.S., Walsh K.B., Naiker M. (2021). Measurement of Gingerols and 6-Shogaol in Ginger Using Near-Infrared Spectroscopy. Proceedings of the The International Conference on NIR.

[39] Moghaddasi M.S., Kashani H.H. (2012). Ginger (*Zingiber officinale*): A Review. J. Med. Plants Res..

[40] Jalali-Jivan M., Nejatian M., Fathi M., Rezaei A., McClements D.J., Jafari S.M. (2025). Different Delivery Systems for Improving the Bioavailability of Ginger Bioactive Compounds: A Comprehensive Review. Phytochem. Rev..

[41] Gonzalez-Gonzalez M., Yerena-Prieto B.J., Carrera Fernández C.A., Vázquez Espinosa M., González-de-Peredo A.V., Garcia Alvarado M.A., Palma Lovillo M., del Carmen Rodríguez-Jimenes G., Fernández Barbero G. (2023). Determination of Gingerols and Shogaols Content from Ginger (*Zingiber officinale* Rosc.) through Microwave-Assisted Extraction. Agronomy.

[42] Ayustaningwarno F., Anjani G., Ayu A.M., Fogliano V. (2024). A Critical Review of Ginger’s (*Zingiber officinale*) Antioxidant, Anti-Inflammatory, and Immunomodulatory Activities. Front. Nutr..

[43] Tohma H., Gülçin İ., Bursal E., Gören A.C., Alwasel S.H., Köksal E. (2017). Antioxidant Activity and Phenolic Compounds of Ginger (*Zingiber officinale* Rosc.) Determined by HPLC-MS/MS. J. Food Meas. Charact..

[44] Juárez U. (2019). Comparación de Dos Técnicas de Extracción de Jengibre (*Zingiber officinale* Roscoe) y Cuantificación de Fenólicos Totales y Capacidad Antioxidante. Investig. Desarro. Cienc. Tecnol. Aliment..

[45] Ezez D., Tefera M. (2021). Effects of Solvents on Total Phenolic Content and Antioxidant Activity of Ginger Extracts. J. Chem..

[46] Jorge-Montalvo P., Vílchez-Perales C., Visitación-Figueroa L. (2023). Evaluation of Antioxidant Capacity, Structure, and Surface Morphology of Ginger (*Zingiber officinale*) Using Different Extraction Methods. Heliyon.

[47] Numan E.M., Jyad J.S., Alazawi A.H., Ibrahim A.-J., Essam F., Jwad A.N., Zehrawo H.M., Kamel A.F., Khayri O.A. (2016). Comparison of different extraction methods of (*Zingiber officinale*) on chemical composition, antioxidant activity. Int. J. Pharm. Pharm. Sci..

[48] Quispe B.D.L.C., Pujaico R.I.B.Q. (2020). Comparación Del Contenido de Compuestos Fenólicos y Capacidad Antioxidante de Los Extractos Hidroalcohólicos de *Zingiber officinale* (Jengibre) Colectados En Tres Zonas de Cultivo En El Departamento de Junín. Bachelor’s Thesis.

[49] Stoilova I., Krastanov A., Stoyanova A., Denev P., Gargova S. (2007). Antioxidant Activity of a Ginger Extract (*Zingiber officinale*). Food Chem..

[50] Hussain G., Saeed F., Shahbaz M., Ahmed A., Imran M., Khan M.A., Faiz F., Bano Y., Munir R., Nadeem M. (2019). Reconnoitring the Impact of Different Extraction Techniques on Ginger Bioactive Moieties Extraction, Antioxidant Characterization and Physicochemical Properties for Their Therapeutic Effect. Pak. J. Pharm. Sci..

[51] Mustafa I., Chin N.L. (2023). Antioxidant Properties of Dried Ginger (*Zingiber officinale* Roscoe) Var. Bentong. Foods.

[52] Ghasemzadeh A., Jaafar H.Z.E., Rahmat A. (2010). Antioxidant Activities, Total Phenolics and Flavonoids Content in Two Varieties of Malaysia Young Ginger (*Zingiber officinale* Roscoe). Molecules.

[53] Ghasemzadeh A., Jaafar H.Z.E., Baghdadi A., Tayebi-Meigooni A. (2018). Formation of 6-, 8- and 10-Shogaol in Ginger through Application of Different Drying Methods: Altered Antioxidant and Antimicrobial Activity. Molecules.

[54] Höferl M., Stoilova I., Wanner J., Schmidt E., Jirovetz L., Trifonova D., Stanchev V., Krastanov A. (2015). Composition and Comprehensive Antioxidant Activity of Ginger (*Zingiber officinale*) Essential Oil from Ecuador. Nat. Prod. Commun..

[55] Grabsk A.H.A., Avincola A.S., Claus T., Porto C., Visentainer J.V., Pilau E.J. (2017). Direct Incorporation of Ginger and Oregano Antioxidants in Canola Oil. J. Braz. Chem. Soc..

[56] Taskeen S., Hafeez M.A., Ikram I. (2025). Investigating the Role of Ginger Tea ORAC Value in Hypertension Management. Insights-J. Health Rehabil..

[57] Pulido Torres S.A., Tamara Angulo G., Hernández Carrión M. (2021). Comparación Entre Dos Materiales de Pared Para la Encapsulación de Extracto de Jengibre Orientado a la Producción de Una Bebida Anti-resaca.

[58] Zagórska J., Pietrzak K., Kukula-Koch W., Czop M., Laszuk J., Koch W. (2023). Influence of Diet on the Bioavailability of Active Components from *Zingiber officinale* Using an in Vitro Digestion Model. Foods.

[59] Contreras-López E., Castañeda-Ovando A., Jaimez-Ordaz J., del Socorro Cruz-Cansino N., González-Olivares L.G., Rodríguez-Martínez J.S., Ramírez-Godínez J. (2020). Release of Antioxidant Compounds of *Zingiber officinale* by Ultrasound-Assisted Aqueous Extraction and Evaluation of Their In Vitro Bioaccessibility. Appl. Sci..

[60] Szymczak J., Grygiel-Górniak B., Cielecka-Piontek J. (2024). *Zingiber officinale* Roscoe: The Antiarthritic Potential of a Popular Spice—Preclinical and Clinical Evidence. Nutrients.

[61] Songvut P., Nakareangrit W., Cholpraipimolrat W., Kwangjai J., Worasuttayangkurn L., Watcharasit P., Satayavivad J. (2024). Unraveling the Interconversion Pharmacokinetics and Oral Bioavailability of the Major Ginger Constituents: [6]-Gingerol, [6]-Shogaol, and Zingerone after Single-Dose Administration in Rats. Front. Pharmacol..

[62] Shaker N.O., El-Naggar M.E., El-Sawey M.M., El-Rahman H.N.A. (2013). Bioactivity of Ginger, *Zingiber officinale* Rhizomes Extract against Two-Spotted Spider Mite, Tetranychus Urticae Koch (Acari: Tetranychidae) and Characterization of Its Volatile Components Using GC/MS Technique. J. Plant Prot. Pathol..

[63] Blank-Landeshammer B., Klanert G., Mitter L., Turisser S., Nusser N., König A., Iken M., Weghuber J. (2022). Improved Bioavailability and Bioaccessibility of Lutein and Isoflavones in Cultured Cells In Vitro Through Interaction with Ginger, Curcuma and Black Pepper Extracts. Antioxidants.

